# An Emotion Aware Task Automation Architecture Based on Semantic Technologies for Smart Offices

**DOI:** 10.3390/s18051499

**Published:** 2018-05-10

**Authors:** Sergio Muñoz, Oscar Araque, J. Fernando Sánchez-Rada, Carlos A. Iglesias

**Affiliations:** Intelligent Systems Group, Universidad Politécnica de Madrid, 28040 Madrid, Spain; o.araque@upm.es (O.A.); jf.sanchez@upm.es (J.F.S.-R.); carlosangel.iglesias@upm.es (C.A.I.)

**Keywords:** ambient intelligence, smart office, emotion regulation, task automation, semantic technologies

## Abstract

The evolution of the Internet of Things leads to new opportunities for the contemporary notion of smart offices, where employees can benefit from automation to maximize their productivity and performance. However, although extensive research has been dedicated to analyze the impact of workers’ emotions on their job performance, there is still a lack of pervasive environments that take into account emotional behaviour. In addition, integrating new components in smart environments is not straightforward. To face these challenges, this article proposes an architecture for emotion aware automation platforms based on semantic event-driven rules to automate the adaptation of the workplace to the employee’s needs. The main contributions of this paper are: (i) the design of an emotion aware automation platform architecture for smart offices; (ii) the semantic modelling of the system; and (iii) the implementation and evaluation of the proposed architecture in a real scenario.

## 1. Introduction

The emergence of Internet of Things (IoT) opens endless possibilities for the Information and Communication Technologies (ICT) sector, allowing new services and applications to leverage the interconnection of physical and virtual realms [1]. One of these opportunities is the application of Ambient Intelligence (AmI) principles to the workplace, which results in the notion of smart offices. Smart offices can be defined as “*workplaces that proactively, but sensibly, support people in their daily work*” [2].

A large body of research has been carried out on the impact that emotions have on decision making [3], health [4], emergencies [5] and working life [6]. This states the importance of recognizing and processing the emotions of people in intelligent environments. Particularly in the workplace, emotions play a key role, since the emotional state of workers directly affects other workers [7] and, consequently, company business. The application of emotion aware technologies to IoT environments entails a quantitative improvement in the workers’ quality of life, since it allows the environment to be adaptive to these emotions and, therefore, to human needs [8]. In addition, this improvement in worker quality of life directly affects company performance and productivity [9].

Emotion Aware AmI (AmE) extends the notion of intelligent environments to detect, process and adapt intelligent environments to users’ emotional state, exploiting theories from psychology and social sciences for the analysis of human emotional context. Considering emotions in the user context can improve customization of services in AmI scenarios and help users to improve their emotional intelligence [10]. However, emotion technologies are rarely addressed within AmI systems and have been frequently ignored [10,11].

A popular approach to interconnect and personalize both IoT and Internet services is the use of Event-Condition-Action (ECA) rules, also known as trigger–action rules [12]. Several now prominent websites, mobile and desktop applications feature this rule-based task automation model, such as IFTTT (https://ifttt.com/) or Zapier (https://zapier.com/). These systems, so-called Task Automation Services (TASs) [13], are typically web platforms or smartphone applications, which provide an intuitive visual programming environment where inexperienced users seamlessly create and manage their own automations. Although some of these works have been applied to smart environments [14,15], these systems have not been applied yet for regulating users’ emotions in emotion aware environments.

This work proposes a solution that consists in an emotion aware automation platform that enables the automated adaption of smart office environments to the employee’s needs. This platform allows workers to easily create and configure their own automation rules, resulting in a significant improvement of their productivity and performance. A semantic model for the emotion aware TASs based on the Evented Web (EWE) [13] ontology is also proposed, which enables data interoperability and automation portability, and facilitates the integration between tools in large environments. Moreover, several sensors and actuators have been integrated in the system as a source of ambient data or as action performers which interact with the environment. In this way, the design of an emotion aware automation platform architecture for smart offices is the main contribution of this paper, as well as the semantic modelling of the system and its implementation and validation in a real scenario.

The rest of this paper is organized as follows. Firstly, an overview about the related work in smart offices, emotion regulation and semantic technologies is given in Section 2. Section 3 presents the semantic modelling of the system, describing different ontologies and vocabularies which have been used and the relationships between them. Then, Section 4 describes the reference architecture of the proposed emotional aware automation platform, describing the main components and modules as well as its implementation. Section 5 describes the evaluation of the system in a real scenario. Finally, the conclusions drawn from this work are described in Section 6.

## 2. Background

This section describes the background and related work for the architecture proposed in this paper. First, an overview of related work in AmE and specifically in smart offices is given in Section 2.1 and Section 2.2, respectively. Then, the main technologies involved in emotion recognition and regulation are described in Section 2.3 and Section 2.4. Finally, Section 2.5 gives an overview of the state of art regarding to semantic technologies.

### 2.1. Emotion Aware AmI (AmE)

The term AmE was coined by Zhou et al. [16]. AmE is “*a kind of AmI environment facilitating human emotion experiences by providing people with proper emotion services instantly*”. This notion aims at fostering the development of emotion-aware services in pervasive AmI environments.

AmE are usually structured in three building blocks [10,17]: emotion sensing, emotion analysis and emotion services or applications.

**Emotion sensing** is the process of gathering affective data using sensors or auto-reporting techniques. There exists many potential sensor sources, including speech, video, mobile data [18], textual and physiological and biological signals. An interesting research for multimodal sensing in real-time is described in [19]. Then, the **Emotion analysis** module applies emotion recognition techniques (Section 2.4) to classify emotions according to emotion models, being the most popular the categorical and dimensional ones and optionally express the result in an emotion expression language (Section 2.5). **Emotion services or applications** exploit the identified emotions in order to improve user’s life. The main applications are [17] emotion awareness and sharing to improve health and mental well-being to encourage social change [20], mental health tracking [21], behaviour change support [22], urban affective sensing to understand the affective relationships of people towards specific places [23] and emotion regulation [24] (Section 2.4).

The adaptation of AmI frameworks to AmE presents a number of challenges because of the multimodal nature of potential emotion sensors and the need for reducing ambiguity of emotion multimodal sources using fusion techniques. In addition, different emotion models are usually used depending on the nature of the emotion sources and the intended application. According to [25], most existing pervasive systems do not consider a multi-modal emotion-aware approach. As previously mentioned, despite the mushrooming of IoT, there are only few experiences in the development of AmE environments that take into account emotional behaviour, and most of them describe prototypes or proofs of concept [10,11,25,26,27,28,29].

From these works, emotion sensing has been addressed using emotion sources such as speech [25,26,29], text [10], video facial and body expression recognition [24] and physiological signals [24]. Few works have addressed the problem of emotion fusion in AmI [24] where a neural multimodal fusion mechanism is proposed. With regard to regulation techniques, fuzzy [24,29] and neurofuzzy controllers [11] have been proposed. Finally, the fields of application have been smart health [24], intelligent classroom [29] and agent-based group decision making [28].

Even though some of the works mention a semantic modelling approach [10], the reviewed approaches propose or use a semantic schema for modelling emotions. Moreover, the lack of semantic modelling of the AmI platform is challenging for integrating new sensors and adapt them to new scenarios. In addition, these works follow a model of full and transparent automation which could leave users feeling out of control [30], without supporting personalization.

### 2.2. Smart Offices

Although several definitions for smart offices are given in different works [2,31,32], all of them agree in considering a smart office as an environment that supports workers on their daily tasks. These systems use the information collected by different sensors to reason about the environment, and trigger actions which adapt the environment to users’ needs by mean of actuators.

Smart offices should be aligned to the business objectives of the enterprise, and should enable a productive environment that maximizes employee satisfaction and company performance. Thus, smart offices should manage efficiently and proactively the IoT infrastructure deployed in the workplace as well as the enterprise systems. Moreover, smart offices should be able to interact with smartphones and help employees to conciliate their personal and professional communications [33].

Focusing on existing solutions whose main goal is the improvement of workers’ comfort at the office, Shigeta et al. [34] proposed a smart office system that uses a variety of input devices (such as camera and blood flow sensor) in order to recognize workers’ mental and physiological states, and adapts the environment by mean of output devices (such as variable colour light, speaker or aroma generator) for improving workers’ comfort. In addition, HealthyOffice [35] deals with a novel mood recognition framework that is able to identify five intensity levels for eight different types of moods, using Silmee TM device to capture physiological and accelerometer data. Li [36] proposed the design of a smart office system that involves the control of heating, illuminating, lighting, ventilating and reconfiguration of the multi-office and the meeting room. With regard to activity recognition, Jalal et al. [37] proposed a depth-based life logging human activity recognition system designed to recognize the daily activities of elderly people, turning these environments into an intelligent space. These works are clear examples of using smart office solutions for improving quality of life, and they propose systems able to perform environment adaption based on users’ mental state.

Kumar et al. [38] proposed a semantic policy adaptation technique and its applications in the context of smart building setups. It allows users of an application to share and reuse semantic policies amongst them-selves, based on the concept of context interdependency. Alirezaie et al. [39] presented a framework for smart homes able to perform context activity recognition, and proposed also a semantic model for smart homes. With regard to the use of semantic technologies in the smart office context, Coronato et al. [40] proposed a semantic context service that exploits semantic technologies to support smart offices. This service relies on ontologies and rules to classify several typologies of entities present in a smart office (such as services, devices and users) and to infer higher-level context information from low-level information coming from positioning systems and sensors in the physical environments (such as lighting and sound level).

One of the first mentions of emotion sensor was in the form of affective wearables, by Picard et al. [41]. As for semantic emotion sensors, there is an initial work proposed by Gyrard et al. [42]. However, to the extent of our knowledge, there is no work in the literature that properly addresses the topics of emotion sensors and semantic modelling in a unified smart automation platform. This paper aims to fill this gap, proposing a semantic automation platform that also takes into account users’ emotion.

### 2.3. Emotion Recognition

Over the last years, emotion detection represents a significant challenge that is gaining the attention of a great number of researchers. The main goal is the use of different inputs for carrying out the detection and identification of the emotional state of a subject. Emotion recognition opens endless possibilities as it has wide applications in several fields such as health, emergencies, working life, or commercial sector. The traditional approach of detecting emotions through questionnaires answered by the participants does not yield very efficient methods. That is the reason for focusing on automatic emotion detection using multimodal approaches (i.e., facial recognition, speech analysis and biometric data), as well as ensemble of different information sources from the same mode [43].

Algorithms to predict emotions based on facial expressions are mature and considered accurate. Currently, there are two main techniques to realize facial expression recognition depending on its way of extracting feature data: appearance-based features, or geometry-based features [44]. Both techniques have in common the extraction of some features from the images which are fed into a classification system, and differ mainly in the features extracted from the video images and the classification algorithm used [45]. Geometric based techniques find specific features such as the corners of the mouth, eyebrows, etc. and extracts emotional data from them. Otherwise, appearance based extraction techniques describe the texture of the face caused by expressions, and extract emotional data from skin changes [46].

Emotion recognition from speech analysis is an area that is gaining momentum in recent years [47]. Speech features are divided into for main categories: continuous features (pitch, energy, and formants), qualitative features (voice quality, harsh, and breathy), spectral features (Linear Predictive Coefficients (LPC) and Mel Frequency Cepstral Coefficients (MFCC)), and Teager energy operator-based features (TEO-FM-Var and TEO-Auto-Env) [48].

Physiological signals are another data source for recognizing people’s emotions [49]. The idea of wearables that detect the wearer’s affective state dates back to the early days of affective computing [41]. For example, skin conductance changes if the skin is sweaty, which is related to stress situations and other affects. Skin conductance is used as an indicator of arousal, to which it is correlated [50]. A low level of skin conductivity suggests low arousal level. Heart rate is also a physiological signal connected with emotions, as its variability increases with arousal. Generally, heart rate is higher for pleasant and low arousal stimuli compared to unpleasant and high arousal stimuli [50].

### 2.4. Emotion Regulation

Emotion regulation consists in the modification of processes involved in the generation or manifestation of emotion [51], and results an essential component of psychological well-being and successful social functioning. A popular approach to regulate emotions is the use of colour, music or controlled breathing [52,53].

Xin et al. [54,55] demonstrated that colour characteristics such as chroma, hue or lightness produce an impact on emotions. Based on these studies and on the assumption of the power of colour to change mood, Sokolova et al. [52] proposed the use of colour to regulate affect. Participants of this study indicated that pink, red, orange and yellow maximized their feeling of joy, while sadness correlates with dark brown and gray. Ortiz-García-Cervigón et al. [56] proposed an emotion regulation system at home, using RGB LED strips that are adjustable in colour and intensity to control the ambience. This study reveals that warm colours are rated as more tensed, hot, and less preferable for lighting, while cold colours are rated as more pleasant.

With regard to music, several studies [57,58] show that listening to music influences mood and arousal. Van der Zwaag [59] found that listening to preferred music significantly improved performance on high cognitive demand tasks, suggesting that music increases efficiency for cognitive tasks. Therefore, it has been demonstrated that listening to music can influence regulation abilities, arousing certain feelings or helping to cope negative emotions [60]. In addition, it has been demonstrated that different types of music may have different demands on attention [61].

The commented studies show that the adaptation of ambient light colour and music are considerable solutions for regulating emotions in a smart office environment, as this adaptation may improve workers’ mood and increase their productivity and efficiency.

### 2.5. Semantic Modelling

Semantic representation considerably improves interoperability and scalability of the system, as it provides a rich machine-readable format that can be understood, reasoned about, and reused.

To exchange information between independent systems, a set of common rules need to be established, such as expected formats, schemas and expected behaviour. These rules usually take the form of an API (application programming interface). In other words, systems need not only to define **what** they are exchanging (concepts and their relationship), but also **how** they represent this information (representation formats and models). Moreover, although these two aspects need to be in synchrony, they are not unambiguously coupled: knowing how data are encoded does not suffice to know what real concepts the refer to, and vice versa.

The semantic approach addresses this issue by replacing application-centric ad-hoc models and representation formats with a formal definition of the concepts and relationships. These definitions are known as ontologies or vocabularies. Each ontology typically represents one domain in detail, and they borrow concepts from one another whenever necessary [62]. Systems then use parts of several ontologies together to represent the whole breadth of their knowledge. Moreover, each concept and instance (entity) is unambiguously identified. Lastly, the protocols, languages, formats and conventions used to model, publish and exchange semantic information are standardized and well known (SPARQL, RDF, JSON-LD, etc.) [63,64,65].

This work merges two domains: rule-based systems and emotions. We will explore the different options for semantic representation in each domain.

There are plenty of options for modelling and implementing rule-based knowledge, such as RuleML [66], Semantic Web Rule Language (SWRL) [67], Rule Interchange Format (RIF) [68], SPARQL Inferencing Notation (SPIN) [69] and Notation 3 (N3) Logic [70].

EWE [13] is a vocabulary designed to model, in a descriptive approach, the most significant aspects of Task Automation Service (TAS). It has been designed after analyzing some of the most relevant TASs [71] (such as Ifttt, Zapier, Onx, etc.) and provides a common model to define and describe them. Based on a number of identified perspectives (privacy, input/output, configurability, communication, discovery and integration), the main elements of the ontology have been defined, and formalized in an ontology. Moreover, extensive experiments have been developed to transform the automation of these systems into the proposed ontology. Regarding inferences, EWE is based on OWL2 classes and there are implementations of EWE using a SPIN Engine (TopBraid (https://www.w3.org/2001/sw/wiki/TopBraid)) and N3 Logic (EYE (http://eulersharp.sourceforge.net/)).

Four major classes make up the core of EWE: *Channel*, *Event*, *Action* and *Rule*. The class *Channel* defines individuals that either generate *Events*, provide *Actions*, or both. In the smart office context, sensors and actuators such as an emotion detector or a smart light are described as channels, which produce events or provide actions. The class *Event* defines a particular occurrence of a process, and allows users to describe under which conditions should rules be triggered. These conditions are the configuration parameters, and are modelled as input parameters. Event individuals are generated by a certain channel, and usually provide additional details. These additional details are modelled as output parameters, and can be used within rules to customize actions. The recognition of sadness generated by the emotion detector sensor is an example of entity that belongs to this class. The class *Action* defines an operation provided by a channel that is triggered under some conditions. Actions provides effects whose nature depends on itself, and can be configured to react according to the data collected from an event by means of input parameters. Following the smart office context mentioned above, to change the light colour is an example of action generated by the smart light channel. Finally, the class *Rule* defines an *ECA*, triggered by an event that produces the execution of an action. An example of rule is: “*If sadness is detected, then change the light colour*”.

There are also different options for emotion representation. EmotionML [72] is one of the most notable general-purpose emotion annotation and representation languages that offers twelve vocabularies for categories, appraisals, dimensions and action tendencies. However, as shown in previous works [73], the options for semantic representation are limited to a few options, among which we highlight the Human Emotion Ontology (HEO) [74], and Onyx [73], a publicly available ontology for emotion representation. Among these two options, we chose Onyx for several reasons: it is compatible with EmotionML; it tightly integrates with the Provenance Ontology [75], which gives us the ability to reason about the origin of data annotations; and it provides a meta-model for emotions, which enables anyone to publish a new emotion model of their own while remaining semantically valid, thus enabling the separation of representation and psychological models. The latter is of great importance, given the lack of a standard model for emotions. In EmotionML, emotion models are also separated from the language definition. A set of commonly used models is included as part of the vocabularies for EmotionML [76], all of which are included in Onyx.

Moreover, the Onyx model provides a model for emotion conversion, and a set of existing conversions between well known models. Including conversion as part of the model enables the integration of data using different models. Two examples of this would be working with emotion readings from different providers, or fusing information from different modalities (e.g., text and audio), which typically use different models. It also eases a potential migration to a different model in the future.

In addition, Onyx has been extended to cover multimodal annotations [77,78]. Lastly, the Onyx model has been embraced by several projects and promoted by members of the Linked Data Models for Emotion and Sentiment Analysis W3C Community Group [79].

There are three main concepts in the Onyx ontology that are worth explaining, as they are used in the examples in following sections. They are: *Emotion*, *EmotionAnalysis* and *EmotionSet*. They relate to each other in the following way: an EmotionAnalysis process annotates a given entity (e.g., a piece of text or a video segment) with an EmotionSet, and an EmotionSet is in turn comprised of one or more Emotions. Due to the provenance information, it is possible to track the EmotionAnalysis that generated the annotation.

## 3. Semantic Modelling for the Smart Office Environment

**Figure 1:**
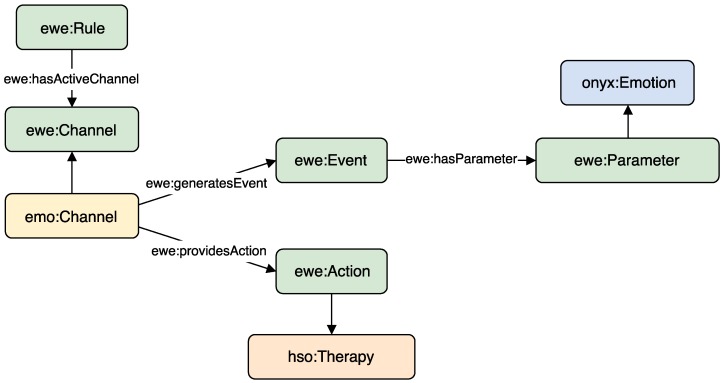
Main classes of the ontologies involved in the semantic modelling.

With the purpose of applying a semantic layer to the emotion aware automation system, several vocabularies and relationships between ontologies have been designed. This enables the semantic modelling of all entities in the smart office environment. Figure 1 shows the relationships between the used ontologies described above.

Automation rules (*ewe:Rule*) are modelled using EWE ontology [13], which presents them in event-condition-action form. Events (*ewe:Event*) and actions (*ewe:Action*) are generated by certain channels. In the proposed architecture, there are different channels that either generate events, provide actions, or both. The class *ewe:Channel* has been subclassed to provide an emotional channel class (*emo:Channel*), which is responsible for generating events and actions related to the emotion recognition and regulation. From this class, the channels *emo:EmotionSensor* and *emo:EmotionRegulator* have been defined. The former is responsible for generating events related to the emotion detection, while the later is responsible for providing certain actions that have the purpose of regulating the emotion. These two classes group all sensors and actuators able to detect or regulate emotions, but should be subclassed by classes representing each device concretely. In addition, events and actions may have parameters. The *emo:EmotionDetected* event has as Parameter the detected emotion. Emotions are modelled using Onyx [73], as described in Section 2.5, so the parameter must subclass *onyx:Emotion*.

The *emo:EmotionRegulator* channel can be subclassed for defining a *SmartSpeaker* or a *SmartLight*, able to provide actions to regulate the emotion such as *emo:PlayRelaxingMusic* or *emo:ChangeAmbientColor*, respectively. The action of playing relaxing music has as parameter (*ewe:Parameter*) the song to be played, while the action of change ambient colour has as parameter the colour to which the light must change. In addition, all these actions are also represented as therapies using Human Stress Ontology (HSO) ontology [80], so *hso:Therapy* has been subclassed. To give a better idea of how specific Channels, Events and Actions have been modelled; Table 1 shows the commented example written in Notation3, describing all its actions with their corresponding parameters.

An example of event and action instances with grounded parameters, which are based on the concepts defined in the listing given above, is presented in Table 2. This table describes the definition of sadness and the actions of playing music and changing ambient colour.

Similarly, automation rules are described using the punning mechanism to attach classes to properties of Rule instances. In the example shown in Table 3, the rule instance describes a rule that is triggered by the event of *sad emotion detection* and produces the action of *changing ambient colour to green* (both defined in Table 2).

## 4. Emotion Aware Task Automation Platform Architecture

**Figure 2:**
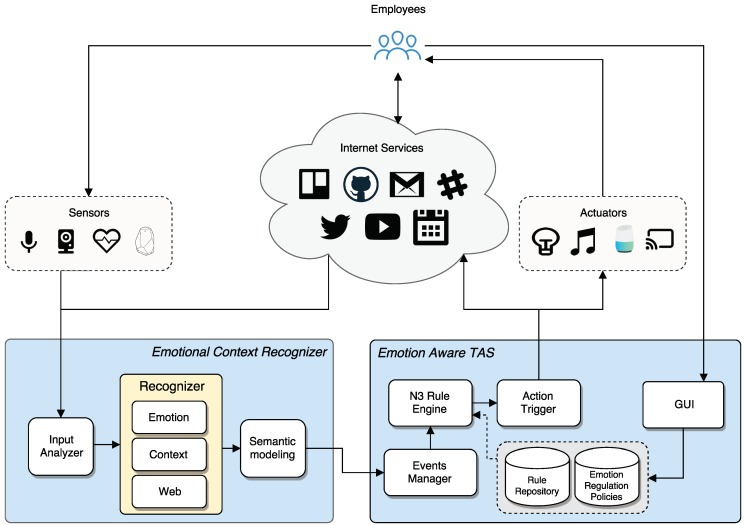
Emotion Aware Automation Platform Architecture.

The proposed architecture was designed based on the reference architecture for TAS [81],which was extended to enable emotion awareness. The system is divided into two main blocks: **emotional context recognizer** and **emotion aware task automation server**, as shown in Figure 2. Emotional context recognizer aims to detect and recognize users’ emotions and information related to context or Internet services and send them to the automation platform to trigger the corresponding actions. The automation system that receives these data is a semantic event-driven platform that receives events from several sources and performs the corresponding actions. In addition, it provides several functions for automating tasks by means of semantic rules and integrates different devices and services.

### 4.1. Emotional Context Recognizer

The emotional context recognizer block is responsible for detecting users’ emotions and contextual events, encoding emotions and events using semantic technologies, and sending these data to the automation platform, where they are evaluated. The block consists of three main modules: input analyzer, recognizer and semantic modelling. In addition, each module is composed of multiple independent and interchangeable sub-modules that provide the required functions, with the purpose of making the system easy to handle.

The input analyzer receives data from sensors involved in emotion and context recognition (such as camera, microphone, wearables or Internet services) and its pre-processing. With this purpose, the input analyzer is connected with the mentioned sensors, and the received data are sent to the recognizer module. The recognizer module receives data captured by the input analyzer. It consists in a pipeline with several submodules that perform different analysis depending on the source of the information. In the proposed architecture, there are three sub-modules: emotion recognizer, context recognizer and web recognizer. The emotion recognizer module provides functions for extracting emotions by means of real time recognition of facial expression, speech and text analysis, and biometric data monitoring; the context recognizer provides functions for extracting context data from sensors (e.g., temperature amd humidity); and the web recognizer provides functions for extracting information from Internet services. Once data have been extracted, they are sent to the semantic modelling module. The main role of semantic modelling is the application of a semantic layer (as described in Section 3), generating the semantic events and sending them to the automation platform.

### 4.2. Emotion Aware Task Automation Server

The automation block consists in an intelligent automation platform based on semantic ECA rules. The main goal is to enable semantic rule automation in a smart environment, allowing the user to configure custom automation rules or to import rules created by other users in an easy way. In addition, it provides integration with several devices and services such as a smart TV, Twitter, Github, etc., as well as an easy way for carrying out new integrations.

The platform handles events coming from different sources and triggers accordingly the corresponding actions generated by the rule engine. In addition, it includes all the functions for managing automation rules and the repositories where rules are stored, as well as functions for creating and editing channels. With this purpose, the developed platform is able to connect with several channels for receiving events, evaluating them together with stored rules and performing the corresponding actions.

To enable the configuration and management of automation rules, the platform provides a *graphical user interface* (GUI) where users can easily create, remove or edit rules. The GUI connects with the rule administration module, which is responsible for handling the corresponding changes in the repositories. There are two repositories in the platform: *rule repository*, where information about rules and channels is stored; and *emotion regulation policies repository*. The policies are sets of rules which aim to regulate the emotion intensity in different contexts. In the smart office context proposed, they are intended to regulate negative emotions to maximize productivity. The rules may be aimed towards automating aspects such as: ambient conditionsto improve the workers’ comfort; work related tasks to improve efficiency; or the rules could adjust work conditions to improve productivity. Some examples of these rules are presented below:

(a) *If stress level of a worker is too high, then reduce his/her task number*. When a very high stress level in a worker has been detected, this rule proposes reducing his/her workload to achieve that his/her stress level falls and his/her productivity rises.
(b) *If temperature rises above 30 °C, then turn on the air conditioning*. To work at high level of temperatures may result in workers’ stress, so this rule proposes to automatically control this temperature in order to prevent high levels of stress.
(c) *If average stress level of workers is too high, then play relaxing music*. If most workers have a high stress value, the company productivity will significantly fall. Thus, this rule proposes to play relaxing music in order to reduce the stress level of workers.

In addition, the company human resources department may implement their own emotion regulation policies to adjust the system to their own context. The system adapts rules based on channel description. Rule adaptation is based on identifying if the smart environment includes the channels used by a certain rule. The system detects available channels of the same channel class used by the rule and request confirmation from the user to included the “adapted rule”. This enables the adaptation of rules to different channel providers, which can be physical sensors (i.e., different beacons) or internet services (i.e., Gmail and Hotmail). The EWE ontology allows us this adaptation by mean of OWL2 punning mechanism for attaching properties to channels [13].

With regards to event reception, these are captured by the *events manager* module, which sends them to the rule engine to be evaluated along with the stored rules. The rule engine module is a semantic engine reasoner [82] based on an ontology model. It is responsible for the reception of events from the *events manager* and the load of rules that are stored in the repository. When a new event is captured and the available rules are loaded, the reasoner runs the ontology model inferences and the actions based on the incoming events and the automation rules are drawn. These actions are sent to the *action trigger*, which connects to the corresponding channels to perform the actions.

The semantic integration of sensors and services is done based on the notion of adapters [83,84], which interact with both sensors and internet services, providing a semantic output. Adapters, as well as mobile clients, are connected to the rule engine through Crossbar.io (https://crossbar.io/), and IoT Middleware that provides both REST-through Web Application Messaging Protocol (WAMP)- and Message Queuing Telemetry Transport (MQTT) interfaces.

Finally, the implementation of this architecture, called EWETasker, was made using PHP for the server, HTML/JavaScript for the web client (including the GUI), and Android SDK for a mobile client. The implementation was based on N3 technology and EYE reasoning engine (http://n3.restdesc.org/). Several sensors and services have already been integrated into EWETasker suitable for the smart office use case. In particular, EWETasker supports indoor and temperature sensors (Estimote bluetooth beacons (https://estimote.com)), smart object sensor (Estimote bluetooth stickers), electronic door control based on Arduino, video emotion sensors (based on Emotion Research Lab), social network emotion sensor (Twitter), and mobile-phone sensors (Bluetooth, location, wifi, etc.). With regards to corporate services, several services oriented to software consultancy firms have been integrated for collaboration (Twitter, GMail, Google Calendar, and Telegram) and software development (Restyaboard Scrum board (http://www.restya.com), GitHub (https://github.com) and Slack (https://slack.com)).

## 5. Experimentation

As already stated, the main experimental contribution of this work was the design and implementation of an emotion aware automation platform for smart offices. In this way, we raised four hypotheses regarding the effectiveness of the proposed system:

- H1: The use of the proposed platform regulates the emotional state of a user that is under stressful conditions.
- H2: The actions taken by the proposed platform do not disturb the workflow of the user.
- H3: The use of the proposed system improves user performance.
- H4: The use of the system increases user satisfaction.

To evaluate the proposed system with respect to these hypotheses, an experiment with real users was performed. For this experiment, a prototype of the proposed system was deployed, which includes the following components. The emotion of the participants was detected from a webcam feed, which feeds a video-based emotion recognizer. As for the semantic layers of the system, the events manager, rule engine and action trigger were fully deployed. Finally, the actuators implemented both hearing and visual signals using a variety of devices. Detailed information on materials is given in Section 5.2. This section covers the design, results and conclusions drawn from the experiment, focusing on its scope.

### 5.1. Participants

The experiment included 28 participants. Their ages ranged from 18 to 28 years, all of them university students with technical background, of both genders. All of them were unaware of this work, and no information regarding the nature of the experiment was given to the participants beforehand. Since the proposed system is primarily oriented to technical work positions, this selection is oriented to validate the system with participants that are currently working in technical environments, or will in the future.

### 5.2. Materials

The material used for this experiment is varied, as the proposed automation system needs several devices to properly function. Regarding the deployment of the automation system, the TAS ran in a commodity desktop computer, with sufficient CPU and memory for its execution. The same environment was prepared for the emotion recognizer system. For the sensors and actuators, the following were used:

- Emotion Research software (https://emotionresearchlab.com/). This module provides facial mood detection and emotional metrics that are fed to the automation system. This module is an implementation that performs emotion classification in two main steps: (i) it makes use of Histogram of Oriented Gradients (HOG) features that are used to train with a SVM classifier in order to localize face position in the image; and (ii) the second step consists in a normalization process of the face image, followed by a Multilayer Perceptron that implements the emotion classification. Emotion Research reports 98% accuracy in emotion recognition tasks.
- A camera (Gucee HD92) feeds the video to the emotion recognizer submodule.
- Room lighting (WS2812B LED strip controlled by WeMos ESP8266 board) is used as an actuator on the light level of the room, with the possibility of using several lighting patterns.
- Google Chromecast [85] transmits content in a local computer network.
- LG TV 49UJ651V is used for displaying images.
- Google Home is used for communicating with the user. In this experiment, the system can formulate recommendations to the user.

Participants accessed the web HTML-based interface using a desktop computer with the Firefox browser (https://www.mozilla.org/en-US/firefox/desktop/).

### 5.3. Procedure

During the experiment, each participant performed a task intended to keep the participant busy for approximately 10 min. This task consisted in answering a series of basic math related questions that were presented to the participant via a web interface (e.g., “Solve 24 · 60 · 60”). We used a set of 20 questions of similar difficulty that have been designed so that any participant can answer them within 30 s. The use of a web-based interface allowed us to programmatically perform the session, and to record metrics associated with the experiment.

The workflow of the experiment is as follows. Each participant’s session is divided into two parts. In each part of the session half of the task questions are sequentially prompted to the participant by the examiner system. Simultaneously, the automation system is fed with the information provided by the different sensors that are continually monitoring the participant emotional state. The experiment finishes when all the questions have been answered. In addition, a questionnaire is given to the participants just after the sessions concludes. These questions are oriented to offer the participant’s view of the system. The raised questions are summarized in Table 4. Questions Q2, Q3, Q4 and Q5 are asked twice, once in regard to the no automation part, and the other time in relation to the part with the automation enabled. Questions Q1 and Q2 are designed so that a check of internal consistency is possible; as, if results from these two questions were to disagree, the experiment would be invalid [86].

**Figure 3:**
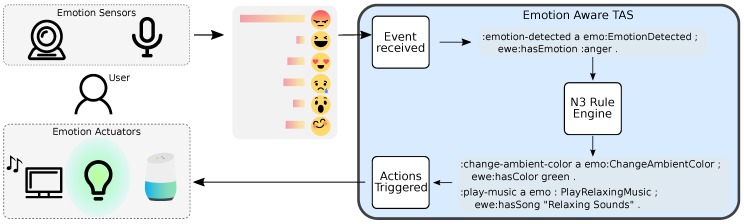
Deployment for the experiment.

The workflow of the system in the context of the experiment is as follows. While the participant is performing the task, the emotion sensor is continuously monitoring the participant’s emotional state. The emotion sensor uses the camera as information input, while the Google Home is used when the user communicates with the system. This emotion-aware data are sent to the TAS, which allows the system the have continuous reports. The TAS receives, processes, and forwards these events to the N3 rule engine. Programmed rules are configured to detect changes in the participant emotional state, acting accordingly. As an example, a shift of emotion, such as the change from happy to sad, is detected by the rule engine which triggers the relaxation actions. If a certain emotion regulation rule is activated, the corresponding action is then triggered through the communication to the action trigger module, which causes the related actuators to start its functioning. The configured actions are aimed at relaxing and regulating the emotion of the participant, so that the performance in the experiment task is improved, as well as the user satisfaction. The actions configured for this experiment are: (i) relaxation recommendations done by the Google Home, such as a recommendation to take a brief walk for two minutes; (ii) lighting patterns using coloured lights that slowly change its intensity and colour; and (iii) relaxing imagery and music that are shown to the user via the TV. A diagram of this deployment is shown in Figure 3.

While the participants are performing the proposed task, the actions of the automation system are controlled. During half of each session, the automation is deactivated, while, during the other half, the action module is enabled. With this, we can control the environmental changes performed by the automation system, allowing its adaptation at will.

Another interesting aspect that could be included is the integration of learning policies based on employee’s emotional state. A related work that models learning policies and their integration with Enterprise Linked Data is detailed in [87].

### 5.4. Design

The experiment was a within-subject design. As previously stated, the controlled factor is the use of the automation system, which has two levels, activated and not activated. The automation use factor is counterbalanced using a Latin square so that the participants are divided into two groups. One group performs the first half of the session without the automation system, while, for the second half of the session, the system is used. The other group performs the task inversely.

### 5.5. Results and Discussion

**Figure 4:**
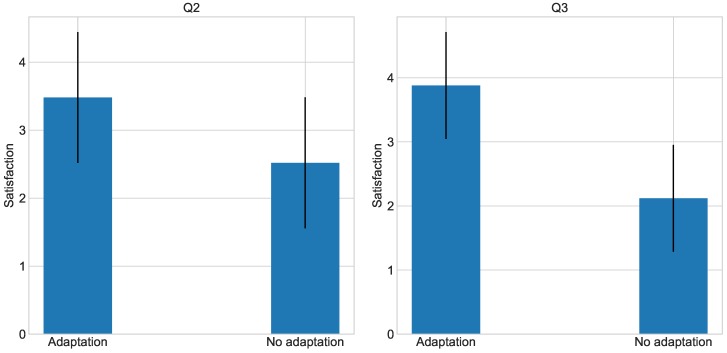
Results for Q2 and Q3.

To tackle Hypothesis 1 and Hypothesis 2, Questions 1 and 2 were analyzed. Regarding Question 1, 18 respondents declared that the section with the adaptation system enabled was the most relaxing for them. In contrast, seven users claimed that for them the most relaxing section of the experiment was that without the adaptation system. The results from Question 1 suggest that users prefer to use the adaptation system, although it seems that this is not the case for all the users. Regarding the Question 2, results show that the average in the adaptation part (3.5) is higher than with no adaptation whatsoever (2.5), as shown in Figure 4. An ANOVA analysis shows that this difference is statistically significant (*p* = 0.015 < 0.05). These results support H1 and H2, concluding that users feel more inclined to use the adaptation system rather than performing the task without adaptation.

Following, Question 3 addressed Hypothesis 3. The analysis of the results of this question reveals that users point higher the usefulness of the environment adaptation for the completion of the task, as shown in Figure 4. While the average for the adaptation section is 3.93, it is 2.07 for the no adaptation part. Through ANOVA, we see that this difference is considerably significant (*p* = 2.96 × 10^−6^ < 0.05). As expected, Hypothesis 3 receives experimental support, indicating that the use of the automation system can improve the performance of the user in a certain task, as perceived by the users.

**Figure 5:**
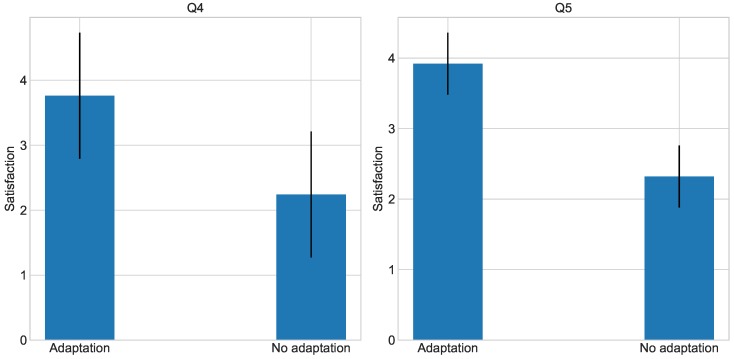
Results for Q4 and Q5.

In relation to Hypothesis 4, both Questions 4 and 5 are aimed to check its validity. As can be seen in Figure 5, users consider more beneficial to work with the adaptation system enabled. The average measure for the adaptation is 3.78, while the no adaptation environment is considered lower on average, with 2.21. Once again, the ANOVA test outputs a significant difference between the two types of environment (*p* = 0.0002 < 0.05). With regard to Question 5, the average for the satisfaction with the adapted environment is 3.83; in contrast, the satisfaction with the no adaptation environment is 2.17, as shown in Figure 5. After performing an ANOVA test, we see that this difference is greatly significant (*p* = 1.02 × 10^−10^ < 0.05). Attending to this, users seem to consider the adaptation system for their personal workspace, and at the same time, they exhibit a higher satisfaction with an adapted work environment. These data indicate that Hypothesis 4 is true, and that users positively consider the use of the adaptation system.

## 6. Conclusions and Outlook

This paper presents the architecture of an emotion aware automation platform based on semantic event-driven rules, to enable the automated adaption of the workplaces to the need of the employees. The proposed architecture allows users to configure their own automation rules based on their emotions to regulate these emotions and improve their wellbeing and productivity. In addition, the architecture is based on semantic event-driven rules, so this article also describes the modelling of all components of the system, thus enabling data interoperability and portability of automations. Finally, the system was implemented and evaluated in a real scenario.

Through the experimentation, we verified a set of hypotheses. In summary: (i) using the proposed automation system helps to regulate the emotional state of users; (ii) adaptations of the automation system do not interrupt the workflow of users; (iii) the proposed system improves user performance in a work environment; and, finally, (iv) the system increases user satisfaction. These results encourage the use and improvement of this kind of automation systems, as they seem to provide users with a number of advantages, such as regulation of stress and emotions, and personalized work spaces.

As future work, there are many lines that can be followed. One of these lines is the application of the proposed system to other scenarios different from smart offices. The high scalability offered by the developed system facilitates the extension of both the architecture and the developed tools with the purpose of giving a more solid solution to a wider range of scenarios. Currently, we are working on its application to e-learning and e-commerce scenarios. In addition, another line of future work is the recognition of the activity, as it is useful to know the activity related to the detected emotion of the user.

Furthermore, we also plan to develop a social simulator system based on emotional agents to simplify the test environment. This system will enable testing different configurations and automations of the smart environment before implementing them in a real scenario, resulting in an important reduction of costs and efforts in the implementation.

## Figures and Tables

**Table 1:** Semantic representation of Emotion Regulator channel written in Notation3.

emo:	SmartSpeaker a owl:Class ;
	rdfs:label “Smart Speaker ” ;
	rdfs:comment “This channel represents a smart speaker .” ;
	rdfs:subClassOf emo:EmotionRegulator .

emo:	PlayRelaxingMusic a owl:Class ;
	rdfs:label “Play relaxing music ” ;
	rdfs:comment “This action will play relaxing music .” ;
	rdfs:subclassOf ewe:Action ;
	rdfs:subclassOf hso:Therapy ;
	rdfs:domain emo:SmartSpeaker .

emo:	SmartLight a owl:Class ;
	rdfs:label “Smart Light ” ;
	rdfs:comment “This channel represents a smart light .” ;
	rdfs:subClassOf emo:EmotionRegulator .

emo:	ChangeAmbientColor a owl:Class ;
	rdfs:label “ Change ambient color ” ;
	rdfs:comment “This action will change ambient color .” ;
	rdfs:subclassOf ewe:Action ;
	rdfs:subclassOf hso:Therapy ;
	rdfs:domain emo:SmartLight .

**Table 2:** Event and action instances.

:sad - emotion - detected a emo:EmotionDetected ;
ewe:hasEmotion onyx:sadness .

:play - music a emo:PlayRelaxingMusic ;
ewe:hasSong “the title of the song to be played ” ;

:change - ambient -color - green a emo:ChangeAmbientColor ;
ewe:hasColor dbpedia:Green .

**Table 3:** Rule instance.

:regulate - stress a ewe:Rule ;
dcterms:title “ Stress regulation rule ”^ xsd:string ;
ewe:triggeredByEvent :sad - emotion - detected ;
ewe:firesAction :change - ambient - color - greenr .

**Table 4:** Questions raised to the participants at the end of the session.

No.	Hypothesis	Question Formulation
Q1	H1, H2	In which section have you been more relaxed?
Q2	H1, H2	What is your comfort level towards the environment?
Q3	H3	Do you think the environment’s state has been of help during the completion of the task?
Q4	H4	Would you consider beneficial to work in this environment?
Q5	H4	What is your overall satisfaction with relation to the environment?
